# Risky sexual behaviour and contraceptive use in contexts of displacement: insights from a cross-sectional survey of female adolescent refugees in Ghana

**DOI:** 10.1186/s12939-019-1031-1

**Published:** 2019-08-16

**Authors:** John Kuumuori Ganle, Doris Amoako, Leonard Baatiema, Muslim Ibrahim

**Affiliations:** 1 Department of Population, Family and Reproductive Health, School of Public Health, University of Ghana, P.O.Box LG 13, Legon, Accra, Ghana; 2 Stellenbosch Institute for Advanced Study (STIAS), Wallenberg Research Centre at Stellenbosch University, Stellenbosch 7600, South Africa; 3 Regional Institute for Population Studies, University of Ghana, Accra, Ghana; 4 Nadowli Hospital, Nadowli, Upper West Region Ghana

**Keywords:** Risky sexual behaviour, contraception, refugee, displacement, female adolescents, global health justice, Ghana

## Abstract

**Background:**

Difficulty in accessing sexual and reproductive healthcare is one of the challenges young refugee women face worldwide, in addition to sexual exploitation, violence and abuse. Although Ghana hosts several refugees, little is known about their sexual behaviour and contraceptive use. This study assesses sexual behaviour and contraceptive use among female adolescent refugees in Ghana.

**Methods:**

A cross-sectional survey was conducted between June and August 2016. Respondents comprised 242 female adolescent refugees aged 14–19 years. Structured validated questionnaires were used to collect data. Descriptive statistical methods and multivariate logistic regression statistical analyses methods were used to analyze data.

**Findings:**

Over 78% of respondents have had penetrative sex; 43% have had coerced sex; 71% have had transactional sex; 36% have had sex while drunk, 57% have had 4–6 sexual partners in the last 12 months before the study, and 38% have had both coerced and transactional sex.

Factors that predicted ever having transactional sex included being aged 14–16 compared to those aged 17–19 (AOR =4·80; 95% CI = 2·55–9·04); being from Liberia compared to being from Ghana (AOR = 3·05; 95% CI = 1.69–13·49); having a mother who had no formal education compared to having a mother with tertiary education (AOR = 5.75; 95CI = 1.94–14.99); and living alone (self) compared to living with parents (AOR = 3.77; 95CI = 1.38–10.33). However, having 1–3 sexual partners in the last 12 months as against having 4–6 partners significantly reduced the odds of ever having transactional sex (AOR = 0·02; 95% CI = 0·01–0·08). Awareness about contraceptives was 65%, while ever use of contraceptives was 12%. However, contraceptive use at last sexual intercourse was 8.2%, and current use was 7.3%. Contraceptive use was relatively higher among those who have never had sex while drunk, as well as among those who have never had transactional sex and coerced sex. Contraceptive use was similarly higher among those who had 1–3 sexual partners in the last 12 months compared to those who had 4–6 during the same time period.

**Conclusion:**

In this time of global migration crises, addressing disparities in knowledge and access to contraception as well as high risk sexual behaviours in refugee situations is important for reducing inequalities in reproductive health outcomes and ensuring both universal health coverage and global health justice. Sex and contraception education and counselling, self-efficacy training, and skills acquisition are needed to help young refugee women negotiate and practice safe sex and resist sexual pressures.

## Introduction

Worldwide, some 68.5 million individuals were forcibly displaced in 2017 as a result of persecution, conflict or generalised violence [1]. Of this number, 25.4million were refugees; 40 million were internally displaced people; and 3.1 million were asylum-seekers [1]. Low-income countries hosted 85% of the world’s refugees under the mandate of the United Nations High Commissioner for Refugees (UNHCR) [1]. With 6.3 million refugees, Sub-Saharan Africa (SSA) hosted about one-third of the world’s refugee population in 2017 [1]. The number of refugees in SSA increased by 1.1 million (22%) during 2017 owing mainly to the crisis in South Sudan [1].

Girls and women constituted about 51% of refugees and internally displaced persons in SSA in 2017 [1]. In addition to food and nutrition security challenges that female refugees face, literature suggest that they are often at high risk of rape, unwanted pregnancies, and sexually transmitted infections [2]. Currently, there are limited national-level statistics on modern contraceptive use among refugee populations in many low-income settings [3]. However, there is evidence that many women in refugee situations face serious reproductive health challenges [3–6]. One of such challenges is unintended pregnancy, which the UNHCR has identified as a crucial reproductive health issue in crises situations [7]. Indeed, living in a refugee situation could increase young girls' and women’s vulnerability to unintended pregnancies and other reproductive health risks in a variety of ways [3, 7–9]. These include early sexual debut; taking sexual risks such as multiple sexual partnership and having intercourse without using a condom; and facing exploitation in the absence of traditional socio-cultural constraints. Moreover, in displacement situations, young refugee women may be forced to resort to risky sexual behaviours, such as prostitution and transactional sex in order to survive [7–9]. This is largely because displacements are often accompanied by poverty, powerlessness and loss of security [8]. In such circumstances, use of modern contraceptives has been reported to be lowest for young refugee women [2–6, 10, 11]. Other previous studies have found significant knowledge gaps in understanding and use of modern contraceptives among refugee populations [8–11].

Globally, several studies have been conducted to examine contraceptive use and determinants among adolescent girls and women in non-refugee situations [12–17]. Few empirical studies have however been undertaken to understand the sexual behaviours, and contraceptive needs and behaviours among female adolescent refugees [18]. In particular, there is scarce information on risky sexual behaviours and contraceptive use among adolescent refugees in settled camp environments [3–11, 18, 19]. In Ghana for example, recent data suggest that awareness about modern contraceptives is universal (> 99%) among girls and women in the general population, and approximately 1 in 3 (27%) married women are using any contraceptive method [20]. However, there are currently no published studies on modern contraceptive use among female adolescent refugees in Ghana, even though there are more than 42,000 refugees in the country [21]. Much of the existing research does not address the perspectives of refugees [18]. This knowledge gap could potentially hinder effective planning and delivery of reproductive health services to women living in refugee situations. Although it is important to identify and address the contraceptive needs and challenges of all women, studies that focus on such hard-to-reach populations as female adolescent refugees are particularly important. Such studies are particularly critical at this time of global refugee crises for building more inclusive reproductive healthcare services that have the potential to improve equity in health outcomes. The purpose of this study was to assess sexual behaviour and modern contraceptive use among female adolescent refugees in the Budumburam refugee camp in Ghana.

## Materials and methods

### Study design

A cross-sectional survey was conducted, using validated structured questionnaires as data collection instruments. The design, implementation and reporting of results followed the Strengthening the Reporting of Observational Studies in Epidemiology (STROBE) checklist for cross-sectional studies.

### Study context

The Budumburam Refugee Camp was the study’s site. The Camp is located in the Central Region of Ghana, 44 km west of Accra [22]. The UNHCR, together with the Ghana Refugee Board (GRB), opened the Camp in 1990, and it is currently the largest of four refugee camps in Ghana [21]. The camp is home to some 42,000 refugees and internally displaced persons [21]. Most refugees are from Liberia, who fled their country during the First Liberian Civil War (1989–1996) and the Second Liberian Civil War (1999–2003) [21]. Refugees from Sierra Leone who also escaped from the civil war (1991–2001) are present. Refugees from Ivory Coast and other internally displaced Ghanaians also live in the camp. The camp however is not a highly restricted zone, and this allows mixing of refugees and non-refugees on regular basis.

In terms of healthcare, the St Gregory Catholic hospital, also known as the Budumburam hospital, is the only hospital at the camp [22]. Sexual and reproductive health services, including family planning and contraception counselling and services, are generally available as part of broader maternal and child health services offered at the hospital. There is however considerable pressure on the services offered at the Budumburam hospital. Consequently, other auxiliary service providers such as community pharmacies often serve as important sources of contraceptive information and services.

### Study population

Female adolescent refugees aged 14–19 years were included in the study. To be included, a respondent aged 14–19 must have been resident (irrespective of years of residence) in the camp, and be registered with the GRB as a refugee or internally displaced person.

### Sample size, sampling and recruitment procedures

To determine a minimum sample size that could allow for any significant statistical association between independent variables and the outcome variable to be detected, we assumed that 19% of the adolescent refugees have ever used modern contraceptives. This assumption is based on contraceptive use prevalence among adolescents reported in Ghana’s most recent demographic and health survey [20]. Based on this assumed prevalence of modern contraceptive use, and assuming a confidence level of 95%, a statistical power of 80%, and a 5% margin of error, we estimated a minimum sample size of 227 using Cochran’s statistical formula [23]. To ensure that the stud was powered enough however, we aimed to include all young refugee adolescents who met our inclusion criteria. To do this, we approached the GRB to request for data covering all female refugees and internally displaced persons aged 14–19 years at the time we approached the Board. The GRB has a database covering nearly all refugees and internally displaced persons in the Camp. A total of 322 potential respondents were obtained after the researchers were given access to the entire database to screen all registered refugees and internally displaced persons in the camp. All the 322 female adolescent refugees/internally displaced persons were included in the study. Following this initial screening and identification of potential respondents, we made several recognizance visits to the camp to identify these adolescents. The identification process started with initial engagement with Camp managers, and country representatives of refugee associations. Management of the hospital was also engaged in this initial process. This engagement gave the researchers an opportunity to explain the purpose of the research to camp managers and leaders and to gain their support. Following this engagement, leaders of various refugee associations helped the researchers to identify the respondents. A total of 238 adolescents were successfully identified through this process. Some 84 potential participants could not be traced for a variety of reasons, including relocation outside the camp and back to their home country. However, a total of eight (8) additional potential respondents were identified. These were not initially part of the list the Board gave the researchers access. In all, 246 female adolescent refugees/internally displaced persons were included as the final sample size.

Finally, the researchers visited each of the selected adolescents in the camp, where the purpose of the study and sampling procedures were thoroughly explained. They were then given one week to decide on their participation if they were alone or aged 18+ or discuss their participation in the study with their parents/guardians/partners if they were living with one and aged below 18 years. They were each re-contacted via telephone after the one-week period. Where the decision was in favour of participation, survey dates were arranged. However, where the decision was against participation (and there were only four such cases), such adolescents were dropped from the study.

### Ethical considerations

Empirical research involving human subjects, particularly vulnerable groups like adolescent refugees, is a moral and an emotional encounter much as it is a scientific and intellectual enterprise. This makes the process of data collection and analysis dialectic, between moral judgment and intellectual rigour. Therefore, issues of ethics must be taken seriously. For this reason, the protocol for this study was submitted to the Ghana Health Service Ethical Review Committee for ethical review and approval (ETHICS APPROVAL–ID NO.: GHS-ERC:12/12/2015). In addition, written permission to conduct the study was obtained from the Ghana Refugee Board, and UNHCR.

#### Informed consent

Before each potential study respondent was surveyed, she signed or thumb-printed a written consent form after detailed explanations. Consent of parents/guardians were obtained for respondents below 18 years, and such respondents then assented to their parents’/guardians’ consent. The informed consent form contained names and telephone numbers of the Principal Investigator and the administrator of the Ghana Health Service Ethical Review Committee. Prior to all surveys*,* the interviewers reviewed the informed consent form with each respondent. Respondents were particularly told about the rational of the study, the procedures and amount of time they will be required to spend on answering survey questions. Also, the benefits and risks of the study and how they were selected to take part in the study were communicated to each respondent. All such information was presented or communicated in a language that was understandable to each respondent. A copy of the signed or thumb-printed consent form was given to the participant and another one kept by the lead investigator for future reference.

#### Voluntary participation and right to withdrawal

Respondents were told individually that participation in the study was purely voluntary, and that they were free not to participate in the study (even if their parent/guardian had given consent), withdraw consent at any time, and refuse to answer any question in the course of the survey. Respondents were also informed that their decision not to participate in the study will not have any negative consequences for them or their families.

#### Confidentiality

The confidentiality of all study respondents was protected. Respondents were not identified by name on any survey questionnaire or any other documentation. All study records were also kept in a locked file cabinet. All computer entry and networking programmes only identified respondents with coded identification numbers. Respondents were also not reported by name in any report or publication resulting from data collected in this study.

#### Privacy

The privacy of all respondents was ensured. Surveys were conducted in venues that ensured maximum privacy of respondents in addition to being convenient to every respondent. Neither the name/address of respondents nor any voice identifiers were used to identify individual respondents. Data from this research were entered into access-controlled and password protected databases, accessible only to the research team, and members of the Ghana Health Service Ethical Review Committee based upon request.

#### Benefits

All respondents were informed that there were no direct benefits of participating in this study. They were however informed that the information that they will each provide may help improve access to sexual, reproductive and maternal healthcare services for women and girls living under refugee situations. Also, no compensation was paid to respondents for participating in the research. However, transport reimbursement was provided to those who travelled to take part in the survey.

#### Risks

No biological samples were collected, and respondents were not exposed to any physical danger when they took part in the study apart from the time they spent answering the questions. However, some respondents did feel uncomfortable discussing their sexual health issues, especially in contexts where sexual abuse had occurred. In such situations, arrangements were made and respondents were referred to appropriate health facilities or healthcare providers for psychological counselling and support. Where the psychological counselling and support services came with cost, the PI provided appropriate financial support to help defray the cost of such services.

### Data collection methods and instruments

Structured questionnaires were designed and administered to collect data. The questionnaire included validated questions on contraceptive knowledge and use from the Ghana Demographic and Health Survey 2014 [20]. Relevant additional questions were included based on previous studies in Tanzania [6], Kenya [7], and Finland [3]. To avoid any misinterpretations of questions and to further validate the instrument, draft questionnaires were pre-tested in one of the three other smaller refugee camps not included in this study. We tested the reliability of the instrument and realised a Cronbach’s alpha coefficient of 0.89. This level of reliability of our data collection tool is considered in literature to be good [24].

The actual data were collected between June and August 2016. Interviews were conducted in three languages: English, French and *Twi* (the most commonly spoken local dialect in Ghana).

### Data entry and processing

The administered questionnaires were first manually examined for completeness, then hand–coded and entered into Microsoft Excel. To ensure data quality, the second and third authors independently entered the data. The first author then compared the two data entries. Errors and inconsistencies that were detected were discussed and resolved before a single database was created and exported into Stata 14 version software for further cleaning. Cleaning of the data was done by running frequencies on each variable. This checked inconsistently coded data. All inconsistently coded data were double-checked with raw data from the questionnaire, and all inconsistencies and errors were resolved.

### Variables

The main outcome variable was ever use of modern contraceptive. We defined modern contraceptive use in line with the Ghana Demographic and Health Survey as the use of any of these methods: female sterilisation, male sterilisation, intrauterine device (IUD), implants, injectable, the pill, male condoms and female condoms, and lactational and amenorrhoea method (LAM) [20]. This was measured as a categorical variable with a dichotomous outcome, and coded as 1 if respondent has ever used any of the modern contraceptive methods above, and 0 if the respondent has not. A secondary outcome variable was risky sexual behaviour, which we broadly defined to include multiple sexual partnerships, drunk sex, coerced sex (e.g. rape), and transactional sex (that is, trading sex for food, protection or other material and psychosocial benefits). We measured multiple sexual partnership in terms of the number of sexual partners a respondent has had in the last 12 months before the survey. The measurement was originally done using an ordinal scale (i.e. 1, 2, 3 tec.), but we recoded this ordinal scale into an interval scale as 1 if the respondent had 1–3 sexual partners in the last 12 months before the survey, and 2 if the respondent had 4–6 sexual partners. Drunk sex was measured as a categorical variable with a dichotomous outcome, and coded as 1 if respondent has ever had sex while drunk, and 0 if the respondent has not. Coerced sex was also measured as a categorical variable with a dichotomous outcome, and coded as 1 if respondent has ever had coerced sex, and 0 if the respondent has not. Furthermore, transactional sex was measured as a categorical variable with a dichotomous outcome, and coded as 1 if respondent has ever traded sex for food, protection or other material and psychosocial benefits, and 0 if the respondent has not.

Several independent variables were also measured, including socio-demographic factors like age and education; sexual behavioural factors like age at first sexual debut; health facility and community level factors such as knowledge of places to get contraceptives.

### Statistical analysis

Categorical variables were summarised into frequencies and proportions. Continuous variables were summarised into mean and range. We checked for the skewedness of the distribution of the sample as well as all variables, and all were normally distributed. Socio-demographic characteristics of respondents, risky sexual behaviours and use of modern contraceptives were summarised using descriptive statistics. Bivariate analyses (i.e. chi-square test of independence and fishers exact test) were first performed to examine association between a total of 26 socio-demographic, knowledge/awareness, perception, health system, behavioral and socio-cultural factors, and modern contraceptive use on the one hand, and ever having transactional sex on the other hand. Following from this bivariate analysis, binary and multiple logistic regression models were fitted and odds ratios were estimated to further assess the strength of association among variables that were significantly associated with ever having transactional sex at the bivariate level. For the multivariate analysis, we followed a combination of two approaches to include variables in the multivariate regression model. First, we followed a data-driven approach, where variables that showed statistical association in the bivariate analysis were included in the multivariate model as potential covariates. Second, we also followed a theory-driven approach, where we drew on theoretical literature that suggested there could be a direct relationship either between an independent variable and the outcome variable of interest or that one independent variable could potentially confound the association between another independent variable and the outcome of interest. Confidence level was held at 95%, and *P* < 0·05 was considered statistically significant.

## Results

### Sample Characteristics

Out of the 246 respondents planned for the study, questionnaires were successfully completed for 242 respondents, giving a response rate of 98·4%. Table 1 shows the background characteristics of respondents. Mean age was 16·8 years (SD = + 2.1). Majority (66·9%) were aged 14–16 years. Some 8·3% of the respondents have never been to school. Majority (90·9%) were unmarried. Majority (83·5%) were Christians. Majority (68%) also lived with their parents.

**Table 1:** Demographic characteristics of respondents

Characteristic	Frequency	Percent
Age		
14–16	162	66.9
17–19	80	33.1
Country of birth		
Liberia	71	29.3
Sierra Leone	11	4.5
Ivory Coast	99	40.9
Ghana	28	11.6
Other	33	13.7
Level of education		
None	20	8.3
Primary	68	28.1
Junior High School	75	31.0
Secondary	57	23.6
Tertiary	18	7.4
Other	4	1.7
Marital status		
Married	22	9.1
Single	220	90.9
Occupation		
Student	210	86.8
Self – Employed	32	13.2
Religious affiliation		
Christianity	203	83.9
Islamic	39	16.1
Person respondent lives with		
Parents	164	67.8
Guardian	35	14.5
Partner	15	6.2
By myself	28	11.6
Highest level of education of mother		
None	76	31.4
Primary	45	18.6
Junior High School	69	28.5
Secondary	33	16.6
Tertiary	19	7.8
Highest level of education of father		
None	58	24.0
Primary	45	18.6
Junior High School	48	19.8
Secondary	53	21.9
Tertiary	38	15.7
Occupation of father		
Self-employed	154	63.6
Unemployed	54	22.3
Government worker	34	14.1
Occupation of mother		
Self-employed	164	68.1
Unemployed	63	26.1
Government worker	14	5.8

### Sexual behaviours

Sexual behaviour among respondents is shown in Table 2. Some 78·2% of the respondents have ever had penetrative sex. Age at first sexual debut ranged from < 10 years (7·3%) to between 15 and 19 years (64·2%). Among those who have had penetrative sex, 43% have had coerced sex; 71% have had transactional sex; 36% have had sex while drunk; 57% have had 4–6 sexual partners in the last 12 months before the study, and 38% have had both coerced and transactional sex.

**Table 2:** Sexual behaviour among young refugee women in Budumburam refugee camp

Statement	Frequency	Percent
Ever had sex before (*n* = 242)^a^		
Yes	193	78.2
No	49	21.8
Ever had coerced sex (rape/forced) (*n* = 193)^b^		
Yes	53	27.5
No	140	72.5
Ever had transactional sex (sex for goods/services) (*n* = 193)^b^		
Yes	137	70.9
No	56	29.1
Age at first sexual debut (*n* = 193)^b^		
< 10	14	7.3
10–14	55	28.5
15–19	124	64.2
Currently have any sexual partner (*n* = 193)^b^		
Yes	150	77.7
No	43	22.3
Ever had sexual intercourse while drunk (*n* = 193)^b^		
Yes	19	9.5
No	174	90.5
Number of sexual partners in the last 12 months (*n* = 193)^b^		
1–3	83	43.0
4–6	110	57.0
Pressured to have sexual intercourse (unmarried =220)^c^		
Yes	188	85.5
No	32	14.5
Source of Pressure****		
Adult men	152	62.8
Friends (boys)	129	53.4
Friends (girls)	43	17.8
Had any sex education in school (*n* = 242)^a^		
Yes	52	21.45
No	170	70.25
Not Applicable	20	8.3
Had any sex education in house (*n* = 242)^a^		
Yes	37	15.29
No	205	84.71
Had any sex education in church/mosque (*n* = 242)^a^		
Yes	12	4.96
No	214	88.43
Not Applicable	16	6.61

^a^This question was asked to all the 242 respondents

^b^This question was asked to only respondents who have ever had sex (i.e. 193), since our interest is understanding sexual behaviour

^c^We excluded the 22 married respondents since sexual intercourse is generally expected in a marital relationship; therefore, the issue of pressure may not apply

^d^Multiple response were allowed

Table 3 also shows results of multivariate logistic regression analyses examining factors that predict ever having had transactional sex.

**Table 3:** Factors associated with ever having transactional sex among sexually active female adolescent refugees (*n* = 193)

Characteristic	Crude OR (95% CI)	Adjusted OR (95%CI)
Age^a^		
17–19*(ref)*	1	1
14–16	6.17 (3.42–11.15)*	4.80 (2.55–9.04)*
Country of birth^b^		
Ghana *(ref)*	1	1
Liberia	2.53 (1.67–9.45)*	3.05 (1.69–13.49)*
Ivory Coast	1.25 (0.52–3.02)	0.87 (0.31–2.41)
Sierra Leone	0.6 (0.31–1.13)	0.68 (0.33–1.39)
Other	0.19 (0.43–1.10)	0.23 (0.06–1.81)
Mothers educational status^c^		
Tertiary *(ref)*	1	1
Secondary	0.44 (0.15–1.23)	0.73 (0.22–2.42)
JHS	0.25 (0.08–0.74)	0.42 (0.12–1.43)
Primary	1.02 (0.36–2.85)	0.83 (0.27–2.49)
None	9.70 (1.76–24.20)*	5.75 (1.94–14.99)*
Person living with^d^		
Parents *(ref)*	1	1
Partner	1.46 (0.67–3.20)	1.12 (0.47–2.62)
Guardian	2.46 (0.84–7.19)	1.99 (0.59–6.69)
Self	7.03 (2.88–14.14)*	3.77 (1.38–10.33)*
Number of sexual partners in the last 12 months^e^		
4–6 *(ref)*	1	
1–3	0.03 (0.02–0.07)*	0.02 (0.01–0.08)*
Knows a place in the community to get contraceptive^f^		
No *(ref)*	1	1
Yes	0.52 (0.29–0.96)*	0.20 (0.30–1.27)
Thinks one unprotected sex can result in pregnancy^g^		
No *(ref)*	1	1
Yes	0.98 (0.40–2.37)	0.50 (0.42–3.12)
Thinks modern contraceptive can offer 100% protection against pregnancy^h^		
Yes *(ref)*	1	1
No	0.32 (0.15–0.68)	0.20 (0.08–0.48)
Ever used modern contraceptives		
Yes *(ref)*	1	1
No	20.3 (6.90–59.70)*	14.70 (4.60–47.20)*
Ever had sex while drunk^j^		
No *(ref)*	1	1
Yes	8.63 (3.50–21.30)*	6.77 (1.16–17.50)*

OR = odds ratio; CI = confidence interval; *ref* = reference category; *p < 0.05

^a^Adjusted for country of birth, mothers educational status, person living with, number of sexual partners in last 12 months, thinks one unprotected sex can result in pregnancy, thinks contraceptives can offer 100% protection against pregnancy, ever use of modern contraceptives, and ever had sex while drunk; ^b^Adjusted for age, mothers educational status, person living with, number of sexual partners in last 12 months, thinks one unprotected sex can result in pregnancy, thinks contraceptives can offer 100% protection against pregnancy, ever use of modern contraceptives, and ever had sex while drunk; ^c^Adjusted for age, country of birth, person living with, number of sexual partners in last 12 months, thinks one unprotected sex can result in pregnancy, thinks contraceptives can offer 100% protection against pregnancy, ever use of modern contraceptives, and ever had sex while drunk; ^d^Adjusted for age, country of birth, mothers educational status, number of sexual partners in last 12 months, thinks one unprotected sex can result in pregnancy, thinks contraceptives can offer 100% protection against pregnancy, and thinks women using contraceptives become promiscuous; ^e^Adjusted for age, country of birth, mothers educational status, person living with, thinks one unprotected sex can result in pregnancy, thinks contraceptives can offer 100% protection against pregnancy, ever use of modern contraceptives, and ever had sex while drunk; ^f^Adjusted for age, country of birth, mothers educational status, person living with, number of sexual partners in last 12 months, thinks contraceptives can offer 100% protection against pregnancy, ever use of modern contraceptives, and ever had sex while drunk; ^g^Adjusted for age, country of birth, mothers educational status, person living with, number of sexual partners in last 12 months, thinks contraceptives can offer 100% protection against pregnancy, ever use of modern contraceptives, and ever had sex while drunk; ^h^Adjusted for age, country of birth, mothers educational status, person living with, number of sexual partners in last 12 months, knowing a place to get contraception, thinks one unprotected sex can result in pregnancy, ever use of modern contraceptives, and ever had sex while drunk; ^i^Adjusted for age, country of birth, mothers educational status, person living with, number of sexual partners in last 12 months, knowing a place to get contraception, thinks one unprotected sex can result in pregnancy, thinks contraceptives can offer 100% protection against pregnancy, ever use of modern contraceptives, and ever had sex while drunk; and ^j^Adjusted for age, country of birth, mothers educational status, person living with, number of sexual partners in last 12 months, knowing a place to get contraception, thinks one unprotected sex can result in pregnancy, thinks contraceptives can offer 100% protection against pregnancy, ever use of modern contraceptives, and ever had sex while drunk

The adjusted odds of ever having transactional sex were higher for respondents who were aged 14–16 compared to those who were aged 17–19 (AOR =4·80; 95% CI = 2·55–9·04). Respondents from Liberia also had significantly higher adjusted odds of ever having had transactional sex compared to respondents from Ghana (AOR = 3·05; 95%CI = 1.69–13·49). Similarly, those whose mothers had no formal education had significantly higher odds of having ever engaged in transactional sex compared to those whose mothers had tertiary education (AOR = 5.75; 95CI = 1.94–14.99). Again, respondents who lived alone (self) had significantly higher odds of reporting ever engaging in transactional sex compared to those who lived with their parents (AOR = 3.77; 95CI = 1.38–10.33). However, having 1–3 sexual partners in the last 12 months as against 4–6 significantly reduced the odds of ever having transactional sex (AOR = 0·02; 95% CI = 0·01–0·08).

### Awareness, perceptions and use of modern contraceptives

More than half (64·5%) of the respondents had heard about contraceptives (Table 4). Male condom (54·8%), pills (22·8%) and implants (19·2%) were the most mentioned methods (not shown here). Respondents’ most important source of information on contraception included television (41·7%), radio (27·6%) and health workers (11·5%). However, 38·5% of the respondents who had heard about contraceptives did not know any specific place where they could get contraception. For those who reported knowing a specific place, community pharmacy shops (48·1%) and friends (35·9%) were the commonly mentioned places.

**Table 4:** Awareness, knowledge and use of contraceptives among young refugee women in Budumburam refugee camp

Characteristic	Frequency	Percent
Ever heard about modern contraceptives (*n* = 242)		
Yes	156	64.5
No	86	35.5
Ever used any modern contraceptive (*n* = 193)		
Yes	23	11.7
No	170	88.3
Use of contraceptive at last sex (*n* = 193)		
Yes	16	8.2
No	177	91.8
Current use of contraceptive use (*n* = 193)		
Yes	14	7.3
No	179	92.7
Thinks contraceptive can give 100% protection against pregnancy (*n* = 156)		
Yes	30	19.8
No	75	47.9
Don’t know	51	32.3
Knows specific place to get modern contraceptive (*n* = 156)*		
Hospital	17	10.9
Pharmacy	75	48.1
Family planning clinic	28	17.9
Friend	56	35.9
Don’t know	76	48.7
Thinks contraception is a woman’s business (*n* = 156)		
Yes	129	82.6
No	27	17.4
Thinks women who use contraceptives become promiscuous (*n* = 156)		
Yes	131	83.5
No	25	16.5

*Multiple responses allowed

In terms of contraceptive use, only 11·7% of respondents who reported ever having sex have ever used modern contraceptives. This reduced to 8·2% during last sexual act. However, only 7.3% of sexually active respondents reported currently using a modern contraceptive method.

As only a few of the respondents reported ever or current use of contraception, it was not statistically prudent to perform regression analysis to examine associations. However, further analysis of the data (not shown here) shows that ever use of contraceptive varied among different categories of respondents. For example, contraceptive use was relatively higher among those aged 17–19 compared with those aged 14–16. Similarly, contraceptive use was relatively higher among those who have never had sex while drunk compared to respondents who have ever had sex while drunk, as well as among those who have never had transactional sex and coerced sex. Contraceptive use was also higher among those who had 1–3 sexual partners in the last 12 months compared to those who had 4–6 during the same time period. Also, contraceptive use was higher among those who reported knowing a place to get contraceptives in the community compared to those who said they did not know any place.

In addition, we examined the links between sex education in school, house, church/mosque and contraceptive use, as well as the link between having a belief that women who use modern contraceptives become promiscuous, and actual use of modern contraceptives. The results are shown in Table 5. Generally, those who reported having had sex education in church/mosque as well as in the house were more likely to report modern contraceptive use compared to those who did not. Those who reported having sex education in school did not report more contraceptive use than those who reported that they did not receive sex education in school. Finally, respondents who reported that woman who use modern contraceptives become promiscuous were far less likely to report modern contraceptive use compared to those who said no (6.7% Vs. 62.9%).

**Table 5:** Percentage distribution of modern contraceptive use by selected respondents’ characteristics

Characteristic	Contraceptive Use, n(%)	
	No	Yes
Thinks women who use modern contraceptive become promiscuous (*n* = 156)*		
Yes	(90.3)	4 (6.7)
No	(37.1)	19 (62.9)
Had any sex education in church/mosque (*n* = 242)**		
Yes	1 (8.3)	11 (91.7)
No	210 (98.1)	4 (1.9)
Not Applicable	8 (50.0)	8 (50.0)
Had any sex education in school (*n* = 242)**		
Yes	34 (65.4)	18 (34.6)
No	168 (98.8)	2 (1.2)
Not Applicable	17 (85.0)	3 (15.0)
Had any sex education in house (*n* = 242)**		
Yes	17 (45.9)	20 (54.1)
No	202 (98.5)	3 (1.5)

*This question applied to only the 156 respondents who have ever heard about modern contraceptives

**This question applied to all 242 respondents in the study

## Discussion

This study is one of the first in Ghana to assess sexual behaviour and modern contraceptive use among female adolescent refugees. Results suggested that majority of respondents were sexually active and engaged in potentially high-risk sexual behaviour such as multiple sexual partnership and transactional sex. However, only 12% have ever used a modern contraceptive.

Many of the findings of this study raise issues that should not be ignored. To begin with, both awareness about modern contraceptives and use among female adolescent refugees in this study are far lower than the over 99 and 19% reported, respectively, for awareness and use among non-refugee adolescents in Ghana [20]. Indeed, the awareness level in this study is lower than levels reported in similar previous studies [6, 10, 18]. There is therefore a need to target adolescent refugees in the camp with educational interventions to increase awareness and knowledge about contraceptives, contraceptive commodities and services. Of course, awareness is only one part of what is needed. Increasing access to, and use of contraceptives is also important, especially given the high percentage of respondents who reported transactional sex. Given that healthcare provision in the camp may not be effective, there is a need for the Ghana Health Service, Ghana Refugee Board and UNHCR to team up and develop outreach services such as mobile clinics to reach these adolescent girls in the camp with both information and services.

The age at first sexual debut for some respondents in this study was less than 10 years. Early sexual debut is a widely reported phenomenon in many parts of Africa [25–27]. However, the possibility of unplanned adolescent pregnancy and sexually transmitted infections (STIs) is a source of concern in this study. This is particularly so for female adolescent refugees who may be unprepared biologically, emotionally and economically to deal with adverse outcomes like teen pregnancy. This could lead to several adverse health outcomes, including unsafe abortion, which accounts for about 20.8% of maternal deaths in some major health facilities in Ghana [28]. In cases where such adolescents contract STIs, they may not even know where to go for treatment. This is potentially concerning given that younger adolescents (14–16 years) in this study had higher odds of engaging in transactional sex. This could potentially contribute to long-term reproductive and non-reproductive health morbidities. Our findings here therefore indicate a need to intensify or implement early sexual and contraception education and counselling interventions for young women in displacement situations. In particular, the Ministry of Education and Ghana Education Service should partner management of the camp to add/ or strengthen sex education in schools. This is particularly important as the school system seems to provide sex-related education to several respondents in this study.

Related to early sexual debut is high-risk sexual behaviours such as multiple sexual partnership and transactional sex. As the results showed, many of the sexually active adolescents (71%) engaged in transactional sex. This is less surprising given that transactional sex in itself may suggest that young girls are already in a disadvantaged material or powerless situation. This is more likely to be the case in refugee situations where poverty, material deprivation and powerlessness are often rife. In this way, sexual activities are likely to be unplanned or where they are planned, young refugee women may lack the bargaining power particularly in the context of transactional sex to negotiate for safe sex. This would suggest that in addition to sex and contraception education and counselling, there is a need for self-efficacy training and skills acquisition to help young refugee women safely negotiate and practice safe sex and/ or resist sexual pressures. It is also important to pay attention to how the common history of trauma among refugees may further complicate decision-making on sexual and reproductive health issues for many female adolescent refugees.

Further, results suggested that respondents aged 17–19 were more likely to use contraceptives compared with those aged 14–16. This is consistent with some studies in non-refugee contexts in Ghana, which suggest that older female adolescents were more than three times likely to practice contraceptive use than younger female adolescents [29]. This age-related disparity could be explained in several ways. Older female adolescents may be more enlightened in terms of knowledge of the benefits of contraception and where to get contraceptives. Also, older adolescents are more likely to be working and be earning income, and may therefore be able both to afford contraceptives on their own, and to negotiate safe sex by insisting on contraceptive use. This is very likely given that respondents aged 17–19 were less likely to engage in transactional sex compared to those aged 14–16. In addition, older female adolescents may have wider social networks from which they could obtain contraceptive information and services than their younger counterparts. This would suggest a need for more targeted interventions like early sexual and contraception education and counselling for younger adolescents. Improved supply of contraceptive commodities at the community level to younger adolescent refugees would also be essential. Given that many of these young female refugees may have experienced sexual and physical violence, it is important that interventions and service delivery occur in socially-safe spaces. These spaces should be easily accessible and less threatening to young adolescent refugees.

Finally, contraceptive use was relatively lesser among respondents who did not know a place to get contraceptives compared to those who knew a place. This finding would suggest a need to go beyond providing education about contraceptives to giving adequate information about appropriate places in the camp and in the wider district/region where contraceptive services may be obtained. Education and information must particularly consider both informational needs and barriers to service uptake such as language barrier. Also, informational and educational programmes should aim to address myths about contraceptive, as several respondents in this study held that women who use contraception may become promiscuous. In this regard, the Ghana Health Service should improve information dissemination and provider-level training to ensure that young refugee women know what to expect when deciding on or approaching service providers for contraception services.

The findings of this study should be interpreted in view of certain limitations. Data on several variables including sexual activity and contraceptive use were retrospectively measured. The possibility of recall bias is therefore acknowledged as it was not possible to independently verify individual responses. Also, it was not possible to explore other aspects like barriers to contraceptive use as well as factors driving high risk sexual behaviours. Further, the study covered only the Budumburam refugee camp. Although we included all potentially eligible female adolescent refugees in the camp, we admit the limitations of generalizing our results beyond the study context. These limitations notwithstanding, important lessons could be learned to inform policy and practice.

## Conclusions

This study provides new information related to sexual behaviour and modern contraceptive use among female adolescent refugees in a settled camp context in Ghana. This information potentially has implications for the sexual and reproductive health needs and rights of girls and women in refugee situations beyond Ghana. Our findings has implications for current screening recommendations from the Center for Disease Control (CDC) and the Office of Refugee Resettlement for those resettling not just in places like the United States but in other African and European contexts. Overall, the results from this study suggest that both awareness about, and use of, modern contraceptives among female adolescent refugees is far lower than the rates among non-refugee adolescents in Ghana. While the disparities in awareness and contraceptive use between female adolescent refugees and their non-refugee counterparts in Ghana could easily be explained, these disparities raise questions about equity, global health justice, and about the reproductive health rights of women living under refugee situations. More important, these disparities could potentially undermine local efforts to achieve equity or universal healthcare access as envisaged in both Ghana’s Health Sector Medium-Term Development Plan (2014–2017) and Ghana’s Millennium Development Goals Acceleration Framework and Country Action Plan. In fact, progress towards attainment of the Sustainable Development Goal 3 covering sexual, reproductive and maternal and child health may not be possible if low contraceptive use of the kind observed in this study is not addressed. There is therefore need for increased attention and resource allocation to provide targeted and evidence-based interventions to both identify sexual and reproductive health needs among women living under refugee situations and address such needs appropriately. Addressing disparities in knowledge, access to, and use of, essential sexual and reproductive health services in refugee situation is particularly important at this time of global refugee crises.

## Data Availability

All relevant data are included in this paper.

## Abbreviations

CDC: Center for Disease Control
GRB: Ghana Refugee Board
IUD: Intrauterine Device
LAM: Lactational Amenorrhoea Method
SSA: Sub-Saharan Africa
STI: Sexually Transmitted Infection
UNHCR: United Nations High Commissioner for Refugees

## References

[1] United Nation High Commissioner for Refugees (2018). Global Treands: Forced Displacements in 2017.

[2] Girard F, Waldman W (2000). Ensuring the reproductive rights of refugees and internally displaced persons: legal and policy issues. Int Perspect Sex Reprod Health.

[3] Degni F, Koivusilta L, Ojanlatva A (2006). Attitudes towards and perceptions about contraceptive use among married refugee women of Somali descent living in Finland. Eur J Contracept Reprod Health Care.

[4] McGinn T, Allen K (2007). Improving refugees’ reproductive health through literacy in Guinea. Glob Public Health.

[5] Whelan A, Blogg J (2007). Halfway people’: Refugee views of reproductive health services. Glob Public Health.

[6] Irani L, Speizer I, Barrington C (2013). Attitudes, beliefs and norms relating to contraceptive use among young migrant and non-migrant adults in urban Dar es Salaam, Tanzania. Glob Public Health.

[7] Women Refugee Commission (WRC) & UNHCR. Baseline study (2011). Documenting knowledge, attitudes and behaviours of Somali refugees and the status of family planning services in UNHCR’s operation in Nairobi, Kenya.

[8] Furuta M, Mori R (2008). Factors affecting women’s health-related behaviours and safe motherhood: a qualitative study from a refugee camp in Eastern Sudan. Healthc Women Int.

[9] Harris C, Symth I (2001). The reproductive health of refugees: lessons beyond ICPD. Gend Dev.

[10] Morrison V (2000). Contraceptive need among cambodian refugees in khao phlu camp. Int Fam Plan Perspect.

[11] Chynoweth (2015). Advancing reproductive health on the humanitarian agenda: the 2012-2014 global review. Confl Heal.

[12] Amalba A, Mogre V, Appiah NAM, Mumuni AW (2014). Awareness , use and associated factors of emergency contraceptive pills among women of reproductive age ( 15–49 years ) in Tamale , Ghana. BMC Womens Health.

[13] Nibabe WT, Mgutshini T (2014). Emergency contraception amongst female college students – knowledge, attitude and practice. Afr J Prim Health Care Fam Med.

[14] Shoveller Jean, Chabot Cathy, Soon Judith A., Levine Marc (2007). Identifying Barriers to Emergency Contraception Use Among Young Women from Various Sociocultural Groups in British Columbia, Canada. Perspectives on Sexual and Reproductive Health.

[15] Farhana I, Karim SI, Ali S, Ali SA (2009). Knowledge of emergency contraception among women of childbearing age at a teaching hospital of Karachi. J Pak Med Assoc.

[16] Marrone G, Abdul-Rahman L, De Coninck Z, Johansson A (2014). Predictors of contraceptive use among female adolescents in Ghana. Afr J Reprod Health.

[17] Galvao L, Diaz J, Diaz M (2015). Emergency Contraception: Knowledge, Attitudes and Practices Among Brazilian Obstetrician-Gynecologists. Int Perspect Sex Reprod Health.

[18] Okanlawon K, Reeves M, Agbaje FO (2010). Contraceptive use: knowledge, perceptions and attitudes of refugee youths in Oru Refugee Camp, Nigeria. Afr J Reprod Health.

[19] Westley Elizabeth, Kapp Nathalie, Palermo Tia, Bleck Jennifer (2013). A review of global access to emergency contraception. International Journal of Gynecology & Obstetrics.

[20] Ghana Statistical Service (GSS), Ghana Health Service (GHS), and ICF International. Ghana Demographic and Health Survey (2014). Rockville, Maryland, USA.

[21] United Nation High Commissioner for Refugees. Ghana Factsheet: July-August 2016*.* Accessed on 8, October, 2017 at http://unhcr-ghana.org/.

[22] Dauda DC (2012). Reproductive Health and Repatriation of Refugee Women in Africa: A Case of Liberian Refugee Women on Buduburam Camp. MA Thesis.

[23] Cochran WG (1977). Sampling Techniques.

[24] Tavakol M, Dennick R (2011). Making sense of Cronbach’s Alpha. Int J Med Educ.

[25] Rijsdijk L, Bos ERA, Lie R (2012). Correlates of delayed sexual intercourse and Condom use among adolescents in Uganda: a cross–sectional study. BMC Public Health.

[26] Eliason S, Awoonor-Williams JK, Eliason C, et al. Determinants of modern family planning useamong women of reproductive age in the Nkwanta district of Ghana: a case–control study. Reprod Health. 2014. http://www.reproductive-health-journal.com/content/11/1/65. 10.1186/1742-4755-11-65. 10.1186/1742-4755-11-65 PMC4274741 25117887

[27] Babatunde OA, Ibirongbe DO, Omede O (2016). Knowledge and use of emergency contraception among students of public secondary schools in Ilorin, Nigeria. Pan Afr Med J.

[28] Der EM, Moyer C, Gyasi RK (2013). Pregnancy related causes of deaths: a 5-year retrospective study. Ghana Med J.

[29] Nyarko SH (2015). Prevalence and correlates of contraceptive use among female adolescents in Ghana. BMC Womens Health.

